# The Effect of Omega-3 Fatty Acids on Psychophysiological Assessment for the Secondary Prevention of Posttraumatic Stress Disorder: An Open-Label Pilot Study

**DOI:** 10.5539/gjhs.v4n1p3

**Published:** 2012-01-01

**Authors:** Kenta Matsumura, Hiroko Noguchi, Daisuke Nishi, Yutaka Matsuoka

**Affiliations:** National Institute of Mental Health, National Centre of Neurology and Psychiatry 4-1-1Ogawa-Higashi, Kodaira 187-8553, Japan Tel: 81-42-346-1986 E-mail: kenta16moon@se.kanazawa-u.ac.jp; National Institute of Mental Health, National Centre of Neurology and Psychiatry 4-1-1Ogawa-Higashi, Kodaira 187-8553, Japan Tel: 81-42-346-1986 E-mail: hi-ro.n@eagle.ocn.ne.jp; Department of Psychiatry and Clinical Research Institute, National Disaster Medical Centre 3256 Midori-cho, Tachikawa, Tokyo 190-0014, Japan Tel: 81-42-526-5511(2309) E-mail: d-nishi@umin.ac.jp; Department of Psychiatry and Clinical Research Institute, National Disaster Medical Centre 3256 Midori-cho, Tachikawa, Tokyo 190-0014, Japan Tel: 81-42-526-5511(2309) E-mail: yutaka@ncnp.go.jp

**Keywords:** Post-traumatic disorder, Fish oil, Fear memory, Script-driven imagery, Startle responses

## Abstract

Our recent pilot study has shown that the supplementation of omega-3 fatty acids (fish oil) immediately after a traumatic event may be effective toward the secondary prevention of post-traumatic disorder (PTSD). To lay the groundwork for addressing the mechanism by which omega-3 fatty acids can prevent PTSD, we analyzed its psychophysiological data. The psychophysiological data included heart rate, skin conductance, and continuous blood pressure during patient subjection to startling tones and idiographic trauma-related cues. Of the 8 patients, 1 met the diagnostic criteria for PTSD. Compared to the seven patients without PTSD, one patient with PTSD showed relatively large reactivity to the startle tones. In contrast, this patient did not show large reactivity to the trauma-related cue during script-driven imagery. The combination of psychophysiological measurements in our randomized control trial should shed light on the underlying mechanisms by which omega-3 fatty acids can prevent PTSD.

## 1. Introduction

We have recently reported that dietary supplementation with omega-3 fatty acids (otherwise known as fish oil) immediately after the occurrence of a traumatic event could be effective in the secondary prevention of post-traumatic disorder (Matsuoka *et al.*, 2010). In this open-label pilot study, the scores of the Clinician-Administered PTSD Scale (CAPS) derived from patients who underwent 12-weeks of omega-3 fatty acids supplementation were significantly lower than their historical scores (Matsuoka *et al.*, 2009).

Although the exact mechanisms by which omega-3 fatty acids can prevent PTSD are not known, we can posit some reasonable hypotheses. One possibility is that the effect is mediated by the omega-3-related facilitation of the clearance of fear memories from the hippocampus (Matsuoka, 2011). Supporting this hypothesis, emerging evidence suggests that omega-3 fatty acids facilitate neurogenesis in the hippocampus(Beltz, Tlusty, Benton, & Sandeman, 2007; Calderon & Kim, 2004) and that such neurogenesis facilitates the clearance of fear memories(Kitamura *et al.*, 2009). Besides this mechanism, a more recent study has revealed that deficits in omega-3 fatty acids can abolish endocannabinoid-mediated neuronal functions (Lafourcade *et al.*, 2011) that facilitate the extinction of fear memories (Marsicano *et al.*, 2002). It is also possible that omega-3 fatty acids are beneficial because of their ability to mediate a reduction of sympathetic activity. A generally accepted notion is that exaggerated and prolonged sympathetic activity contributes to the development of PTSD (Charney, Deutch, Krystal, Southwick, & Davis, 1993); omega-3 fatty acids have been shown to lower this activity(Delarue *et al.*, 2003; Hamazaki *et al.*, 2005; Spence, Thornton, Muir, & Westcott, 2003). However, in any of these cases, a single assessment of CAPS is not sufficient to provide new insights into these potential mechanisms.

In order to lay the groundwork for addressing the mechanism of the effects of omega-3 fatty acids on PTSD, we analyzed psychophysiological data in above-mentioned open-label pilot study. Physiological measurements included heart rate, skin conductance, and blood pressure. The measurement paradigms included assaying patients’ responses to a startling sound and to a trauma-related cue, otherwise known as script-driven imagery. These paradigms are the same as those used to measure autonomic functions, but they quantify different psychophysiological attributes. The indices derived from the first paradigm reflect a mixture of the hyperarousal symptoms(Griffin, 2008; Shalev *et al.*, 2000) and vulnerability(Guthrie & Bryant, 2005; Orr *et al.*, 2003) common to PTSD, while those derived from the second paradigm reflect an inner expression of aversive emotions caused by the recollection of a traumatic event—that is, fear memory(Pitman, Orr, Forgue, de Jong, & Claiborn, 1987). In this study, we report the results of these psychophysiological assessments.

## 2. Methods

### 2.1 Participants

**Figure 1:**
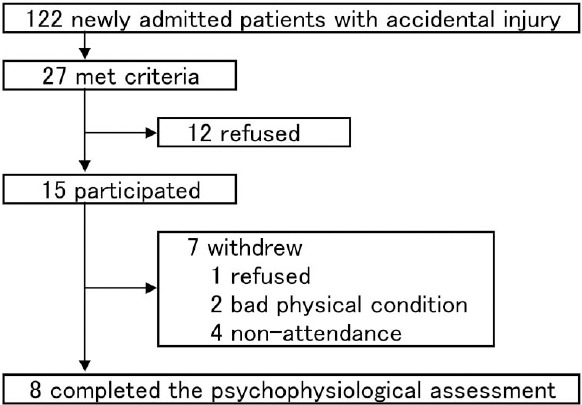
Flowchart of patients

This study was conducted as a part of the Tachikawa Project for Prevention of Post-traumatic Stress Disorder with Polyunsaturated Fatty Acid (registered as NCT00671489 at http://clinicaltrials.gov). All participants were selected from 122 consecutive Japanese patients who were injured and hospitalized into the intensive care unit of the National Disaster Medical Center, Tokyo between May 2008 and October 2008 (see Figure 1). The inclusion criteria were (a) ≥18 years of age, (b) native Japanese-speaking ability, (c) our ability to contact patients within 240 h of injury, and dosing in oral use, and (d) physical and mental ability to understand the scope of the study and to consent to participating in the trial. The exclusion criteria were (a) clearly irretrievable acute brain parenchyma damage and subdural or subarachnoidal bleeding, as detected by computed tomography and/or magnetic resonance imaging, (b) cognitive impairment: mini-mental state examination score < 24, (c) heavy alcohol use or 100 IU/L ≤ γGTP in administration, (d) heavy tobacco use (>40 cigarettes per day), (e) history or current diagnostic suspicion of psychosis or bipolar I disorder, (f) diagnostic suspicion of alcoholic or substance-related disorders or eating disorders, (g) existence of serious symptoms, such as suicidal ideation, self-harm behavior, dissociation, or other status necessitating rapid psychiatric treatment, (h) use of anti-epilepsy drugs, lithium, ethyl icosapentate, or anti-coagulant drugs (for example, aspirin, warfarin) at regular intervals within 3 months of injury, (i) regular use of polyunsaturated fatty acids supplements within 3 months of injury, and (j) a habit of eating fish >4 times a week. Of 27 candidates, 15 agreed to participate in the whole study, but eventually only 8 male patients participated in this psychophysiological study. The reasons for nonparticipation were refusal (*n* = 1), inability to attend due to poor physical condition (*n* = 2), and non-attendance (*n* = 4). The mean age of the participants was 36.6 ± 17.6 years; their accident types were motor vehicle accidents (*n* = 4), fall from a height (*n* = 2), and work-place accidents (*n* = 2). The patients who participated in this study did not differ significantly in age or severity of injury from nonparticipants, but participants tended to be male. Written informed consent was obtained from every participant after providing a complete description of the study. This study was approved by the Institutional Review Board of National Disaster Medical Center.

All participants took a dietary supplement of omega-3 fatty acids containing 1470 mg docosahexaenoic acid and 147 mg eicosapentaenoic acid per day for 12 weeks.

A diagnosis of PTSD was made by the Clinician-Administered PTSD Scale (CAPS; Asukai, Hirohata, Kato, & Konishi, 2003; Blake *et al.*, 1995) at 12 weeks after participation.

### 2.2 Stimuli

The startling tones used in this study were essentially the same as those described in previous studies (Orr, Lasko, Shalev, & Pitman, 1995). Stimuli consisted of 15 sudden, 95 dB(A), 1000 Hz, 500 ms pure tones, with 0 ms rise and fall times. Inter-stimulus intervals were randomly ranged from 27 to 52 s.

Idiographic trauma-related cues used in the study were essentially the same as those described in previous studies(Pitman, *et al.*, 1987), except for language. On the basis of a prior interview by the clinical psychologist, each patient’s experience of their accident was recorded as a traumatic injury script. The script was digitally recorded with a loud, slow, and tense voice by an independent experimenter who had never met the patients before the experiment. The length was approximately 30 s, which corresponds to approximately 160 letters in Japanese.

### 2.3 Apparatus and Physiological measures

A 3 × 3-m, sound-attenuated, temperature-controlled experimental room was used. Stimuli were presented through binaural headphones (Sony, MDR-CD900ST) plugged into USB audio interface (digidesign Mbox2), controlled by Mac OS X computers (Apple, Mac Pro, MA970J/A, Mac OS X 10.5.5). In developing these applications, we included much of the software code utilized in previous studies(Matsumura, Yamakoshi, & Ida, 2009).

Heart rate (HR) was obtained from electrocardiogram (ECG) readings that were recorded through disposable electrodes placed bilaterally on the arms and connected to a bioamplifier (Monte system, ECG100C). Skin conductance (SC) was obtained from the index and middle finger of the left hand through an Ag/AgCl transducer that was filled with isotonic electrode paste and attached to a bioamplifier (Monte system, GSR100C). Mean blood pressure (MBP) was derived from a blood pressure contour. Blood pressure was measured noninvasively through a finger cuff that was attached to the annular finger of the left hand and connected to a continuous blood pressure monitor (MEDi SENS, MUB101). Analog output was sampled at 16 bit, 1000 Hz by an integrated bioamplifier system (Monte system, BIOPAC MP150 system). Output was then transferred and stored digitally on a Mac Pro computer using AcqKnowledge software (Monte system). Software was run in a virtual Windows XP environment (Parallels, Parallels Desktop 4.0) constructed on a Mac Pro.

### 2.4 Procedure

This experiment was conducted after the patients’ diets were supplemented with omega-3 fatty acids for 12-weeks after injury. After administration of initial instructions and setup of instruments, the participants sat in a reclining chair in the experimental room. A 5-min adaptation period was followed by a 10-min startle period. Participants were told to sit quietly and listen to the tones until the experimenter informs them that they are finished.

After a short break, and a second round of instruction, a 3-min adaptation period was followed by a 2-min script period. The script period consisted of 4 continuous 30 s periods, namely: baseline, read, imagery, and recovery. Participants were asked to sit quietly, listen to the script, imagine it as realistically as possible, and relax. To prevent mistakes, short instruction was given to patients immediately before each period, except pre-baseline.

### 2.5 Data Reduction

Startle responses of HR, SC, and MBP were calculated by subtracting the mean HR and SC levels 1 s immediately preceding tone onset from the maximum levels 1 to 4 s after tone onset. Initial MPB levels were similarly subtracted from the MBP maxima that occurred 5 to 8 s after tone onset. These values, except for those from the first trial (Lykken, Iacono, Haroian, McGue, & Bouchard, 1988), were averaged to give a mean response score.

HR, SC, and MBP values during script-driven imagery were averaged over each 30 s period. Then, reactivity during script delivery was calculated by subtracting the baseline level from the imagery level.

## 3. Results

On the basis of CAPS at a 12-weeks-post-injury follow-up examination — that is, immediately after the experiment — 1 patient was diagnosed as having PTSD with a total CAPS score of 76.

### 3.1 Startling tones

**Figure 2:**
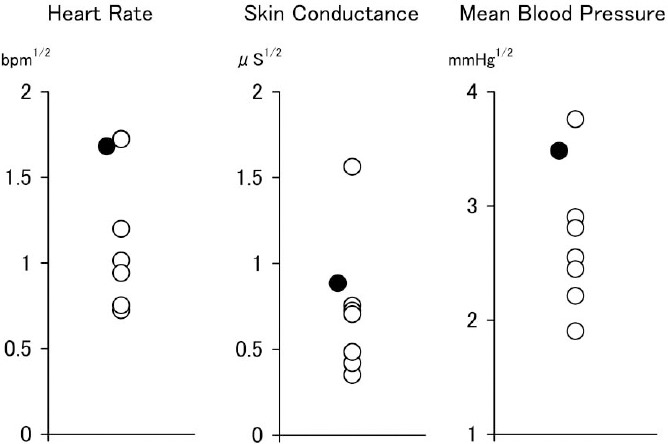
Physiological reactivity to startle sounds in patients with (filled circles) and without (open circles) PTSD after 12-weeks supplementation with omega-3 fatty acids *Note*. BPM = beat per minutes; *μ*S=*μ*Siemens.

Reactivity values of the patient with PTSD and seven patients without PTSD are presented together in Figure 2. Overall, the reactivity of the patient with PTSD was high.

### 3.2 Script-driven imagery

**Figure 3:**
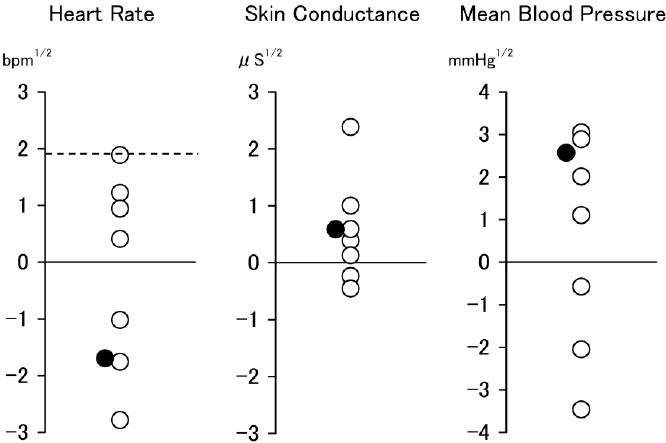
Physiological reactivity during imagery of idiographic trauma-cues in patients with (filled circles) and without (open circles) PTSD after 12-weeks supplementation with omega-3 fatty acids *Note*. Dashed lines represent empirical cut-offs for PTSD. BPM = beat per minutes; *μ*S = *μ* Siemens.

In Figure 3, the reactivity values during imagery of idiographic trauma-cues are presented together with their empirical cut-offs for PTSD for both the patient with PTSD and for the 7 patients without PTSD. The sensitivity and specificity of these cut-offs for PTSD are 69% and 89%, respectively.(Orr, Metzger, & Pitman, 2002) No HR responses exceeded the cut-off (1.9 beats per minutes^1/2^). Additional 1-sample *t*-tests indicated that mean HR^1/2^ reactivity (-0.35 ± 1.69) was significantly below the PTSD cut-off (*t* (7) = 3.77, *p* = .007, *d* = 1.33). Overall, the reactivity of the patient with PTSD was not high.

## 4. Discussion

To begin probing the mechanisms that underlie the prevention of PTSD by omega-3 fatty acids, we conducted a psychophysiological assessment. To our knowledge, no studies have examined the psychophysiology of PTSD in injured Japanese patients. The experimental and measurement procedures were finished without serious difficulty and the raw data obtained had few noticeable artifacts (not shown). It should be noted that, because our laboratory was not barrier-free, patients in wheel chairs or using crutches had some difficulty participating in the experiment.

Reactivity varied according to the measurement paradigm and indices used. The reactivity of the patient with PTSD in the startle tone paradigm (see, Figure 2) was generally large. This tendency is consistent with the previously mentioned notion that startle reactivity reflects a mixture of the hyper-arousal symptoms (Griffin, 2008; Shalev, *et al.*, 2000) and vulnerability (Guthrie & Bryant, 2005; Orr, *et al.*, 2003) often found in PTSD. In contrast, the reactivity in script-driven imagery in the patient with PTSD was not large at all. Considering that reactivity during script-driven imagery reflects inner expression of aversive emotions caused by the recall of traumatic event (Pitman, *et al.*, 1987), this data might suggest that clearance of traumatic event memory—that is, fear memory—from the hippocampus has been facilitated.

We show here the potential usefulness of psychophysiological assessment in examining the underlying mechanisms by which omega-3 fatty acids may prevent PTSD. However, our conclusions are based solely on one patient with PTSD. Therefore, it is currently unknown if such reactivity patterns are representative of PTSD patients in general, or if they are specific to those who have undergone 12 weeks of omega-3 fatty acids supplementation. To examine these questions, we have begun a double-blind, placebo-controlled, randomized trial (registered as NCT00671099 at http://clinicaltrials.gov).

We measured MBP throughout the experiment. To our knowledge, no studies have reported PTSD-related MBP reactivity in a startle paradigm. Because BP increase is observed regardless of patients’ responder type (that is, their tendency to have cardiac or vascular responses; Gregg, Matyas, & James, 2002; Julius, 1988) BP measurements are considered to be one of the most robust indices among all psychophysiological measures. In fact, BP has begun to be regarded as a potentially useful index (Pole, 2007; Tucker *et al.*, 2007). In future studies, BP will be more closely monitored, in addition to the classical measurements of HR and SC.

We confirmed the feasibility of carrying out psychophysiological assessments in patients who have taken omega-3 fatty acids supplements immediately after traumatic events. Psychophysiological assessment appears to be useful in the examination of the mechanisms by which omega-3 fatty acids prevent PTSD.
